# Sphingolipids and Atherosclerosis: The Dual Role of Ceramide and Sphingosine-1-Phosphate

**DOI:** 10.3390/antiox12010143

**Published:** 2023-01-06

**Authors:** Marco Piccoli, Federica Cirillo, Andrea Ghiroldi, Paola Rota, Simona Coviello, Adriana Tarantino, Paolo La Rocca, Ivana Lavota, Pasquale Creo, Paola Signorelli, Carlo Pappone, Luigi Anastasia

**Affiliations:** 1 Laboratory of Stem Cells for Tissue Engineering, IRCCS Policlinico San Donato, Piazza Malan 2, San Donato Milanese, 20097 Milan, Italy; 2 Institute for Molecular and Translational Cardiology (IMTC), San Donato Milanese, 20097 Milan, Italy; 3 Department of Biomedical, Surgical and Dental Sciences, University of Milan, 20133 Milan, Italy; 4 Faculty of Medicine and Surgery, University Vita-Salute San Raffaele, Via Olgettina 58, 20132 Milan, Italy; 5 Department of Biomedical Sciences for Health, University of Milan, Via Mangiagalli 31, 20133 Milan, Italy; 6 Aldo Ravelli Center for Neurotechnology and Experimental Brain Therapeutics, Department of Health Sciences, University of Milan, Via Antonio di Rudinì 8, 20142 Milan, Italy; 7 Arrhythmology Department, IRCCS Policlinico San Donato, Piazza Malan 2, San Donato Milanese, 20097 Milan, Italy

**Keywords:** atherosclerosis, coronary artery disease (cad), sphingolipids, ceramide, sphingosine-1-phosphate, oxidative stress, endothelial dysfunction, atherosclerotic plaque

## Abstract

Sphingolipids are bioactive molecules that play either pro- and anti-atherogenic roles in the formation and maturation of atherosclerotic plaques. Among SLs, ceramide and sphingosine-1-phosphate showed antithetic properties in regulating various molecular mechanisms and have emerged as novel potential targets for regulating the development of atherosclerosis. In particular, maintaining the balance of the so-called ceramide/S1P rheostat is important to prevent the occurrence of endothelial dysfunction, which is the trigger for the entire atherosclerotic process and is strongly associated with increased oxidative stress. In addition, these two sphingolipids, together with many other sphingolipid mediators, are directly involved in the progression of atherogenesis and the formation of atherosclerotic plaques by promoting the oxidation of low-density lipoproteins (LDL) and influencing the vascular smooth muscle cell phenotype. The modulation of ceramide and S1P levels may therefore allow the development of new antioxidant therapies that can prevent or at least impair the onset of atherogenesis, which would ultimately improve the quality of life of patients with coronary artery disease and significantly reduce their mortality.

## 1. Sphingolipids and Atherosclerosis

Sphingolipids (SLs) are a heterogeneous class of complex lipids that contain a common 18-carbon aminoalcohol sphingoid backbone and are known to be essential building blocks of the plasma membrane [1]. Additionally, SLs are highly versatile cellular components. They have increasingly been shown to be critical bioactive molecules dynamically involved in regulating numerous cellular processes, such as signal transduction, differentiation, proliferation, and apoptosis [2]. The physiological functions of SLs are significantly affected by their cellular abundance. In particular, due to the complexity of their metabolism, the potential imbalance between biosynthesis and turnover of SLs may lead to dangerous changes in SL levels and dysfunction. Remarkably, excessive amounts of some SLs, such as ceramides and lyso-ceramides, have been shown to play a crucial role in promoting the pathological mechanism of lipotoxicity, ultimately causing chronic inflammation and deleterious effects on various organs and systems [3]. In this sense, SLs are involved in the pathogenesis of several human diseases, including cancer [4], metabolic disorders [5], cystic fibrosis [6], and cardiovascular disease (CVD) [7]. Among cardiovascular diseases, atherosclerosis (AS) is considered the most common underlying mechanism of these pathologies [8]. It often leads to some of the primary causes of death in the Western world, namely coronary artery disease (CAD), myocardial infarction, and sudden cardiac death. Evidence of a possible link between SLs and atherosclerosis has existed for more than 50 years, when Smith et al. demonstrated that advanced lesions in the intima and media of the vessel wall contained significantly elevated levels of SLs, particularly sphingomyelin (SM) [9]. Interestingly, this initial observation was confirmed by several other studies by independent research groups that identified six specific SLs (SM, dihydroceramides, ceramides, lactosylceramides, glucosylceramides, and sphingosine-1-phosphate) as a typical signature of atherosclerotic plaques, suggesting a direct involvement of this class of molecules in the atherogenic process [10].

Atherogenesis is a chronic and progressive multifocal arterial disease caused by a close interplay between endothelial dysfunction and subendothelial lipoprotein retention in regions of impaired blood flow in medium-sized arteries, the so-called lesion-prone areas [11]. These regions are characterized by a unique pro-inflammatory phenotype, gradually leading to endothelial cell damage. Notably, endothelial dysfunction, which can be considered the trigger of the whole atherogenic process, is also directly involved in the subsequent steps of atherogenesis by influencing plaque development and rupture [12]. Therefore, increased endothelial dysfunction is considered an early indicator of atherogenesis [13]. The underlying cause of endothelial dysfunction is oxidative stress. Indeed, many environmental risk factors for atherogenesis, including cigarette smoking, hypercaloric diet, or pre-existing conditions such as hypercholesterolemia, hyperglycemia, and hypertension, are characterized by uncontrolled production of reactive oxygen species (ROS). Oxidative stress at the endothelium level is due to increased ROS production and decreased availability of nitric oxide (NO). In particular, NADPH oxidases (NOXes) are one of the major sources of cellular reactive oxygen species [14,15,16]. The NOX4 isoform is the most abundant in endothelial cells compared with the other NOX isoforms (i.e., NOX1, NOX2, and NOX5) [15,17,18]. Under physiological conditions, the NOX enzymes produce moderate ROS required for normal redox signaling. In particular, the NOX2 and NOX4 isoforms induce endothelial cell proliferation and survival via ROS -mediated p38 MAPK and Akt activation [19]. Under pathological conditions, NOX4 may promote the formation of a pro-thrombogenic endothelial phenotype [20]. On the other hand, nitric oxide exerts mainly anti-inflammatory and antithrombotic functions, for example, by attenuating platelet adhesion, aggregation, and leukocyte adhesion. Under physiological conditions, nitric oxide is continuously produced and released by endothelial NO synthase (eNOS) [21]. However, in endothelial dysfunction, eNOS produces ROS in a process known as eNOS uncoupling [22], resulting in a deleterious imbalance of ROS that culminates in increased vascular permeability [23]. The disruption of endothelial barrier integrity promoted by endothelial dysfunction allows the extravasation of circulating low-density lipoprotein (LDL) into the intima [24]. In this context, ROS and reactive nitrogen species (RNS) damage the cellular function of lipids, proteins, and carbohydrates and cause lipid peroxidation and low-density lipoprotein (LDL) oxidation [25]. OxLDL is considered the major factor in the development of atherosclerosis. OxLDL can remain in the intima, where they increase the adhesion capacity of leukocytes and induce the expression of leukocyte and monocyte adhesion molecules on the endothelial surface, such as Vascular Cell Adhesion Molecule-1 (VCAM), Intercellular Adhesion Molecule-1 (ICAM), E-selectin, and P-selectins, which cause low-grade inflammation [26,27,28]. Leukocyte recruitment to the arterial wall is a hallmark of atherogenesis and is mediated mainly by two distinct mechanisms. First, as mentioned previously, endothelial dysfunction is associated with the expression of cell adhesion molecules (CAMs), i.e., ICAM-1 and VCAM-1, on endothelial cells [29], and second, with the increased secretion of chemokines that direct monocytes to the atherosclerotic lesion [30].

Most chemotactic molecules originate from circulating platelets and neutrophils or from tissue-resident cells such as endothelial cells and macrophages [31]. In particular, interaction between monocytes and platelets induces reciprocal expression of adhesion molecules and production of chemokines that further promote activation and accumulation of leukocytes in developing lesions [32].

The monocytes accumulated in the subendothelial space start producing ROS, mainly because the damaged endothelial cells and aggregated platelets secrete several pro-inflammatory cytokines such as tumor necrosis factor α (TNF-α) and interleukin 1β, which create an extremely oxidative microenvironment [33]. The latter triggers the oxidation of accumulated LDL to oxidized low-density lipoprotein (oxLDL). These modified LDL can sustain leukocyte recruitment and promote the differentiation of monocytes into phagocytic macrophages, which in turn internalize oxLDL by increasing the expression of atherogenic scavenger receptors such as CD36 and become the so-called foam cells [34]. In addition, activated macrophages and foam cells secrete cytokines and growth factors such as PDGF that induce migration, proliferation, and accumulation of vascular smooth muscle cells (VSMC) from the media into the intima. These VSMCs can internalize oxLDL and develop into foam cells [35,36]. Over time, this process stimulates a non-resolving inflammatory response that promotes the activation of apoptotic and necrotic mechanisms in the foam cells that release their cellular contents [37]. This way, more lipids and cellular debris are incorporated into the dynamic lipid core of the atherosclerotic plaque, leading to its enlargement. As a result, the biomechanical environment and, thus, the stability of the plaque are significantly altered. Indeed, blood flow in the vessels generates axial, circumferential, and shear stress that, in combination, contribute to the overall deformation of the vasculature [38]. The presence of large plaques generates flow obstructions that significantly increase the aforementioned stress, which, in turn, contribute to the instability of plaques [39]. This instability may eventually lead to the destruction of the intima of vulnerable plaques, arterial thrombosis, and end-organ ischemia [34,40]. Therefore, the process of atherogenesis is characterized by a progressive and complex mechanism of increasing damage, in which the pathophysiological role of SLs is crucial. Indeed, many studies have shown that the overall SL metabolism is fundamental in atherogenesis and that different sphingoid mediators play a key role in regulating all the processes underlying this disease.

Among sphingolipids, ceramide and sphingosine-1-phosphate (S1P) in particular are able to differentially regulate cellular functions by modulating opposing signaling pathways. In this context, the reciprocal balance of these two interconnected SLs metabolites has been termed ceramide/S1P rheostat [2]. Recently, major efforts to elucidate the molecular mechanisms and signaling pathways by which these metabolites exert their effects have demonstrated the need to rethink the rheostat concept. Indeed, the regulation of cellular processes is extremely complex and involves many other molecules besides ceramide and S1P [41].

However, ceramide and sphingosine-1-phosphate (S1P) are probably the best characterized SLs as important second messengers. Therefore, this comprehensive review aims to critically summarize their diverse behavior and describe their significant contribution to the development of endothelial dysfunction and progression of atherosclerosis. Moreover, the growing understanding of the involvement of ceramide and S1P in atherogenesis suggests that influencing SL metabolism may pave the way for the development of innovative therapeutic approaches for this disease.

## 2. Ceramide and Sphingosine-1-Phosphate Metabolism

Ceramide is considered the central molecule of SLs metabolism and can be generated via three different biosynthetic pathways: (*i*) the *de novo* synthesis; (*ii*) the sphingomyelinase (SMase) pathway; (*iii*) the catabolic/salvage pathway [42]. Condensation between palmitoyl-CoA and serine by serine palmitoyltransferase (SPT) represents the rate-limiting step in the overall *de novo* synthesis of ceramide. It occurs in the cytosolic leaflet of the endoplasmic reticulum (ER) and generates 3-ketosphinganine. Then, 3-ketosphinganine is reduced by 3-ketosphinganine reductase to generate sphinganine, which is N-acylated by one of the six mammalian isoforms of sphinganine N-acyl transferase, also known as ceramide synthase (CerS1-CerS6), to form dihydroceramide [43]. The final step of the ceramide *de novo* synthesis pathway is the conversion of dihydroceramide to ceramide by introducing a double bond into the sphingosine tail by dihydroceramide desaturase [44]. The importance of this ceramide biosynthetic pathway is supported by evidence that blocking the activity of SPT by myriocin, a natural specific inhibitor of this enzyme, has led to promising treatment in several diseases, including CVD [45,46,47,48,49].

The second route to increase ceramide levels is the sphingomyelinase pathway, which consists of the hydrolysis of the phosphocholine head groups of sphingomyelin (SM) by the activity of sphingomyelinases. Depending on their working pH, these hydrolytic enzymes can be divided into three isoforms: alkaline, acidic, and neutral SMase. Although they catalyze the same reaction, they differ in tissue and subcellular localization [50]. In particular, alkaline SMase expression is restricted to the intestinal mucosa and liver, whereas the acidic and neutral isoforms are ubiquitously expressed throughout the body [51]. In addition, acid sphingomyelinase (aSMase) is mainly located in the endolysosomal compartment but can be secreted into the extracellular space under certain conditions [52]. The secreted isoform of aSMase is directly implicated in the pathogenesis of atherosclerosis, as it has been demonstrated that its release can be promoted by atherogenic proinflammatory cytokines and its activity increases ceramide levels by hydrolysis of SM on lipoprotein particles [53]. Neutral sphingomyelinase (nSMase), on the other hand, has been found mainly in the endoplasmic reticulum and Golgi apparatus but can also translocate to the inner leaflet of plasma membranes [54]. Moreover, nSMase could be also associated to the mitochondrial membrane where they induce ceramide production directly in these organelles [55]. Three different subtypes of nSMase have been identified. Still, only nSMase2 appears to play an essential role in the development of CVD, particularly in promoting endothelial dysfunction and maturation of atherosclerotic plaques [56,57].

Finally, ceramide may be generated by another cellular mechanism, termed either sphingolipid recycling or salvage pathway. This latter mechanism consists of the degradation of complex sphingolipids and involves several key enzymes such as SMases, glucocerebrosidases, and ceramidases that eventually generate sphingosine in the endolysosomal compartment. Finally, sphingosine could be released from lysosomes and reused to produce ceramide by re-acylation mediated by the ceramide synthase mentioned above [58]. Remarkably, the salvage pathway and the formation of sphingosine-1-phosphate (S1P) are closely related. Although sphingosine could be considered the end product of complex SL degradation and reused to form ceramide, it could instead be phosphorylated by sphingosine kinases (SphKs) to generate S1P [59]. SphKs are ubiquitous enzymes in mammals, and two major isoforms have been described, namely SphK1 and SphK2. Although they share the same substrate and catalyze the same phosphorylation, SphK1 and SphK2 have a peculiar tissue distribution. In particular, SphK1 is found primarily in the lung, liver, and spleen, whereas SphK2 is expressed primarily in the liver and heart [60]. Regardless of where it is synthesized, S1P behaves as a bioactive lipid mediator targeting five different G-protein-coupled S1P receptors (S1PR1-5) and is involved in several cellular mechanisms such as proliferation, apoptosis, and migration. In addition, S1P has been linked to the regulation of endothelial barrier function and thus to the pathogenesis of atherosclerosis, leading in particular to atheroprotective effects [61,62].

The ability of ceramide and S1P to activate antithetic signaling pathways is critical for regulating cell fate and maintaining cellular homeostasis. Because these two sphingolipid metabolites are mutually interconvertible, although the respective amounts may be very different, an increase in ceramide levels could simultaneously lead to a decrease in S1P and vice versa. It should be noted, however, that an increase in ceramide concentration is not necessarily accompanied by a decrease in S1P. Indeed, the most important regulator of cellular processes is the balance between these two sphingolipids and not their absolute amount. Although this fundamental concept must be kept in mind, the ceramide/S1P rheostat has emerged as an attractive molecular target for the development of new therapeutic strategies to counteract several diseases, including atherosclerosis.

## 3. Ceramide

The term ceramide refers to a large family of fatty acid derivatives of sphingosine or other related bases, commonly referred to as sphingoid bases [63]. These molecules can be considered structurally as N-acyl amide derivatives of sphingoid bases formed by the condensation of their amino group with the carboxyl group of a fatty acid. The fatty acids are usually saturated or monounsaturated and have chain lengths ranging from 14 to 26 carbon atoms [64]. The heterogeneity of both sphingoid bases and acyl groups has given rise to hundreds of ceramide species with particular physical and chemical properties. Moreover, these structural features are responsible for the distribution of ceramides in specific regions of the plasma membrane (lipid rafts) along with other sphingolipids and receptors, as well as for their ability to act as second messengers and modulate the activity of various proteins involved in many cellular functions such as differentiation, senescence, and apoptosis [64]. The structural complexity of ceramides is reflected in a complex biosynthetic pathway whose critical step is the initial condensation between serine and palmitoyl-CoA. This reaction is catalyzed by SPT, a pyridoxal 50-phosphate-dependent heterodimer composed mainly of two subunits encoded by the ubiquitously expressed genes Sptlc1 and Sptlc2 [65]. However, a third isoform has been identified (Spltc3), which is selectively expressed only in specific tissues such as placenta, skin, and some glands, and is responsible for the synthesis of specific ceramide species [64]. The large heterogeneity of the ceramide family is also due to the different isoforms of ceramide synthases. In mammals, six different subtypes of CerS have been identified, each of which synthesizes ceramides with different acyl chain lengths and has a particular tissue distribution [66]. In particular, CerS1 synthesizes C18-ceramide and is expressed mainly in the brain and in small amounts in skeletal muscle and the testis; CerS2 is specific for ceramides with very long acyl chains and its mRNA is expressed ubiquitously, being particularly abundant in the liver, brain and heart; CerS3 synthesizes C24-ceramides and is found mainly in the skin and testis; CerS4 is involved in the production of ceramide containing C18-22 fatty acids and is expressed at high levels in leukocytes, the heart and liver; CerS5 has been extensively studied for its ability to synthesize the pro-apoptotic C16-ceramide and is expressed in lung epithelium; CerS6 is specific for C14- and C16-ceramides, but is the least characterized of the ceramide synthases [43]. Ceramide production is also mediated by the degradation of complex SLs such as sphingomyelin. The main agents responsible for this degradation are the sphingomyelinases, which, as mentioned earlier, can be divided into three isoforms: alkaline, acidic, and neutral SMase [50]. Apart from their different working pH, these enzymes are expressed in almost all tissues, but with specific subcellular localizations [67]. Therefore, the different nature of ceramide subspecies and the specific subcellular localization of the enzymes involved in their production suggest that ceramide levels may be regulated by different mechanisms in different compartments, thus affecting various cellular processes, including atherosclerosis.

### 3.1. Ceramide and Endothelial Dysfunction

The pathophysiological role of ceramide in atherosclerosis (AS) has been extensively studied [68]. As mentioned before, endothelial dysfunction represents one of the earlier steps in the pathophysiology of atherosclerosis. Endothelial damage results from a complex series of events. Among them, the generation of reactive oxygen species (ROS) and the decreased bioavailability of nitric oxide (NO) appear to be crucial [69,70]. Remarkably, ceramide has been frequently associated with the deleterious effects of ROS (H_2_O_2_, superoxide and hydroxyl radicals) on the endothelium, mainly because ceramide induces their production. Although the molecular mechanisms by which ceramide can increase the levels of ROS are still poorly understood, several evidences associated the cross talk between ceramide and redox signaling to the formation of ceramide-enriched cell membrane microdomains. In this context, these specific regions of the plasma membranes can function as signaling platforms, promoting the recruitment and clustering of different enzymes, such as NADPH oxidase, involved in ROS generation [71].

Conversely, ROS can activate several ceramide-generating mechanisms. In this regard, many reports indicate that various stimuli such as stress, ischemia and reperfusion, and hypoxia can trigger ROS accumulation, which in turn triggers the activation of aSMase, leading to an increase in ceramide levels [72,73,74,75]. Similarly, nSMase activity is stimulated by ROS in various cell types such as vascular smooth muscle cells (VSMCs), fibroblasts, and epithelial cells, ultimately leading to ceramide accumulation [73,76,77]. The molecular mechanisms involved in the ROS-mediated activation of SMases are specific in different cell types [78,79]. However, it is unclear whether and how ROS directly activate ceramide-producing enzymes or inhibit ceramide-degrading proteins but the alteration of the cellular redox balance should represent the major trigger for the activation of these enzymes [80].

The close link between ceramide and ROS is supported by evidence that ceramides, and C16 ceramide in particular, are the major sphingolipids involved in cellular metabolism and energy regulation. Indeed, ceramide increase in mitochondria membranes (specifically short chain and C16-ceramides) may induce permeability and cytochrome C release [81] and may alter the structure and function of the ETC complexes [82,83]. More recently, Vos M. et al. have shown that mitochondrial ceramide accumulation is sufficient to impair ETC, increasing ROS production and promoting mitophagy [84]. However, the mechanisms by which ceramides affect respiratory complex activity are still under investigation.

Ceramides also activate several ROS-generating enzymes such as NADPH oxidase, uncoupled eNOS, and xanthine oxidase in many mammalian cells and animal models [72,85,86]. These effects are essential for endothelial cells, particularly in response to proinflammatory cytokines such as tumor necrosis factor (TNF).

Indeed, treatment of endothelial cells with TNF triggered a rapid increase in mitochondrial ROS production, which was mediated mainly by ceramide-dependent signaling pathways. Specifically, TNF stimulated aSMase, which increased ceramide levels and induced ceramide-activated protein kinases (CAPKs) and ROS production. The CAPKs molecular mechanisms of action still need to be fully clarified. However, they phosphorylate several downstream protein kinases, such as Raf-1 or Rac-1, which in turn activate a protein kinases-mediated signaling pathway, ultimately leading to the activation of ROS-generating enzymes [87,88]. These results were confirmed by complete abrogation of ROS formation by treatment of endothelial cells with the aSMase inhibitor desipramine or with the CAPKs inhibitor DMAP [89]. In addition, Ji Y. et al., recently demonstrated that the aSMase inhibitor amitriptyline counteracts endothelial dysfunction and promotes vascular function. Treatment of TNF-stimulated endothelial cells (ECs) with the inhibitor significantly reduced aSMase activity and ceramide release. Moreover, the beneficial effect of amitriptyline on ECs was mediated by a significant reduction in the formation of ROS, accompanied by an anti-inflammatory effect due to the inhibition of the expression of adhesion molecules promoted by TNF-α [90]. However, the cytotoxic effects of TNF-α on ECs are not exclusively mediated by increasing ceramide levels via the sphingomyelin-ceramide signal transduction cascade through activating sphingomyelinases. Indeed, the *de novo* synthetic pathway may represent an alternative mechanism for ceramide generation upon TNF-α stimulation. This hypothesis is supported by the demonstration that treatment of cerebral artery ECs with fumonisin B1, which is a structural analog of sphingosine and a potent inhibitor of ceramide synthases, protects against TNF-induced cell death [91]. These data, therefore, support the existence of feed-forward mechanisms between inflammation and ceramide that lead to the amplification of redox signaling pathways and the production of ROS, which impairs vascular endothelial homeostasis. When ceramide levels rise to even higher levels, it causes mitochondrial changes that go beyond energetic impairment and lead to cell death. Indeed, elevated C16 ceramide levels promote outer mitochondrial membrane permeabilization (MOMP), which favors cytochrome-c release and the induction of apoptosis [92].

Birbes et al., demonstrated that only mitochondrial overexpression of SMase, which induced accumulation of ceramide at the mitochondrial level, was responsible for cell death. To support these findings, they also showed that upregulation of Bcl-2, one of the major mitochondrial anti-apoptotic factors, prevented the ceramide-induced release of cytochrome c, thereby inhibiting apoptosis. In contrast, overexpression of SMase in other organelles had no effect, suggesting that the action of ceramide is compartmentalized, and each subcellular pool controls specific physiological processes [93].

Moreover, ceramide acted as a second messenger in ionizing radiation-induced endothelial apoptosis. In contrast, cigarette smoke mediated endothelial injury through ROS-dependent upregulation of ceramide, followed by activation of the p38 MAPK and JNK pathways [94,95].

Zhang et al. reported that endothelial dysfunction in coronary arteries is associated with lipid rafts (LR) accumulation and the formation of LR redox signaling platforms [96]. These LR clusters are characterized by increased NADPH oxidase activity in response to various death receptor ligands. Interestingly, some of these ligands stimulate aSMase activity to induce ceramide accumulation, which ultimately promotes the formation of LR clusters by forming ceramide-enriched membrane microdomains. These microdomains could spontaneously fuse and promote the aggregation of NADPH oxidase subunits, stimulating the production of ROS and maintaining the mutual positive feedback loop between ceramide and oxidative signals [97]. More recently, the importance of plasma membrane reorganization has been demonstrated by the formation of ceramide-enriched microdomains in human microvascular endothelial cells (HMEC-1) exposed to ionizing radiation or exogenous C16 ceramide. Such treatments stimulated aSMase activity and increased ceramide levels in the cells, which subsequently underwent significant cytoskeletal restructuring, leading to p38 MAPK activation and apoptosis [98].

Despite the direct link with oxidative stress, endothelial dysfunction could also be characterized by reduced bioavailability of NO resulting from the imbalance between the generation of NO and the degradation of NO due to the production of ROS [99]. While the induction of apoptosis was initially considered the primary mechanism of ceramide-mediated cellular injury [100], ceramide is now associated with regulating many biological processes. Ceramide may influence endothelial activity by directly controlling the NO balance. Indeed, ceramides control vasorelaxation and NO synthesis by dephosphorylation and inactivation of eNOS, upon ceramide-activated PP2A [101]. In addition, *de novo* synthesis of ceramide was shown to be also responsible for eNOS regulation in the endothelium [102]. However, some reports suggest a dual activity of ceramide that could be either protective or detrimental to endothelial cells. Indeed, brief TNF exposure was sufficient to activate nSMase, which increased intracellular ceramide levels and promoted the activation of the PI3K/Akt pathway, leading to eNOS stimulation [103].

Moreover, human endothelial cells treated with exogenous C6 ceramide showed upregulation of both mRNA and protein expression of eNOS. However, the same treatment was also associated with a significant decrease in the bioavailability of NO. Indeed, treating human umbilical vein endothelial cells with ceramide significantly reduced the release of bioactive NO in culture and ceramide levels in intact coronary artery endothelium [86,104]. The discrepancy between the increased expression of eNOS and its decreased activity has been attributed to ceramide’s aforementioned ability to stimulate ROS production, which could lead to the rapid oxidation of NO and, thus, a decrease in its levels [85]. Interestingly, Freed et al., demonstrated that overnight exposure to ceramide in human arterioles was responsible for a shift in vasoactive mediators from NO to mitochondrial hydrogen peroxide (H_2_O_2_) that was completely reversed by the inhibition of nSMase [105]. Although both factors cause vasodilation, they have opposing nonvasomotor effects, as NO is known to trigger anti-inflammatory and antithrombotic signaling pathways, whereas H_2_O_2_ promotes inflammation, thrombosis, and atherosclerosis [106].

Consistent with this observation, Smith et al. demonstrated a link between the age-related increase in nSMase activity and the reduction in eNOS phosphorylation responsible for the loss of synthesis capacity of NO. Specifically, they proposed a ceramide-mediated mechanism which triggers protein phosphatase 2 (PP2A) and ultimately affects the phosphorylation state of eNOS [107]. Recently, this ceramide-mediated mechanism was confirmed in endothelial progenitor cells (EPCs) and isolated vessels exposed to palmitate. The resulting increase in ceramide synthesis was sufficient to activate PP2A and decrease the phosphorylation of Akt and eNOS. Consequently, impairment of the bioavailability of NO negatively affected adherence, migration, and the ability of EPCs to form tubes and ultimately promoted endothelial dysfunction [101,108]. These in vitro results were also confirmed in vivo in mouse and rodent models. Specifically, a high-fat diet markedly increased ceramide levels in mice that developed endothelial dysfunction and exhibited reduced VEGF-mediated aortic eNOS and Akt phosphorylation [109]. In addition, Bharath et al. further elucidated the mechanism between the increase in ceramide levels and the activation of PP2A. Indeed, under physiological conditions, PP2A is retained by inhibitor 2 of PP2A (I2PP2A), which binds phosphatase and keeps it in the cytosol [110]. Ceramide can bind I2PP2A and disrupt its association with PP2A, allowing it to migrate to the plasma membrane. At this subcellular location, PP2A could interact with eNOS and promote dissociation of the Akt-Hsp90-eNOS complex, which is required for eNOS activation [111]. These findings are also corroborated by the fact that inhibition of ceramide *de novo* synthesis by myriocin partially reverses endothelial dysfunction and counteracts the development of atherosclerosis, supporting the hypothesis of a crucial role of ceramide in these pathogenetic processes [101,112].

**Figure 1:**
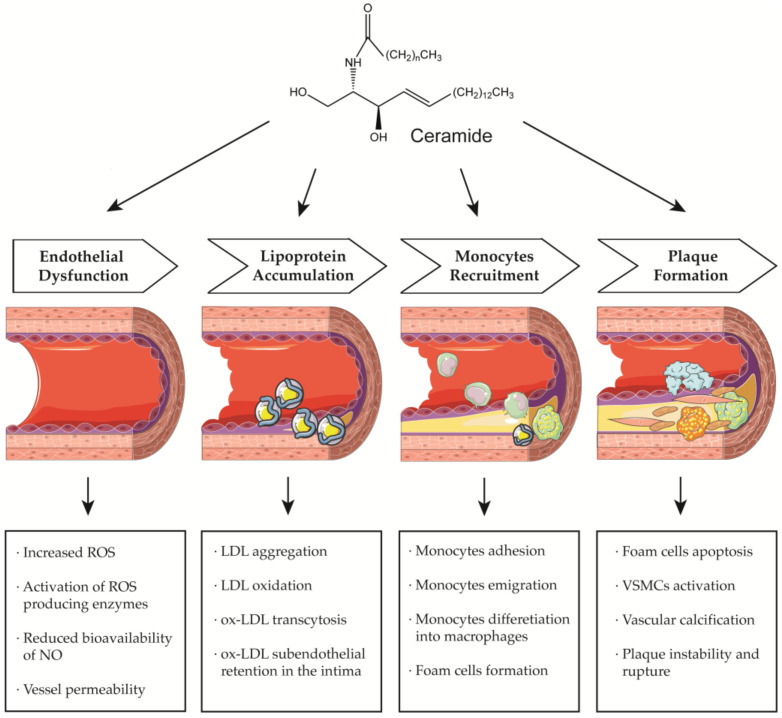
Ceramide involvement in atherosclerotic lesions progression.

More recently, however, Cantalupo et al. have shown that endothelial *de novo* synthesis of this sphingolipid is critical for the maintenance of efficient vascular function and homeostasis. Specifically, although the reduction in ceramide caused by endothelial deletion of the gene Sptlc2, which encodes one of the subunits of SPT, increased basal eNOS phosphorylation and production of NO, it also impaired endothelial signal transduction in response to various chemical stimuli. Moreover, impaired vasorelaxation of Sptlc2-deficient vessels was restored by ceramide administration, particularly C16 ceramide [102]. Thus, these data suggest that ceramide is directly involved in regulating the biological functions of the endothelium (Figure 1). However, further efforts are needed to fully understand how ceramide deregulation might contribute to endothelial homeostasis and dysfunction.

### 3.2. Ceramide and Lipoproteins

Lipoproteins (LPs) are another crucial factor in the development of atherosclerotic lesions, and their role evolves throughout the process. Particularly in the early stages of atherosclerosis, aggregation of atherogenic lipoproteins significantly stimulates the retention of apoB-LP particles, especially low-density lipoprotein (LDL), which are converted to oxidized LDL (oxLDL) and transported through the endothelial cell barrier into lesion-prone areas of the arterial tree [34]. Once in the subendothelium, oxLDL induces the expression of cell adhesion molecules on endothelial cells, promoting the recruitment and accumulation of mainly monocytes and lymphocytes B at sites of arterial injury. At this point, oxLDL may be recognized by activated monocytes and trigger their differentiation into macrophages and subsequently into foam cells. In addition, oxLDL also appears to be responsible for the migration and proliferation of VSMCs, which form a necrotic core around the plaque and enlarge the lesion. Finally, oxLDL are able to induce apoptosis of VSMCs, promoting plaque instability and rupture, which is the worst part of the progression of atherogenesis [113].

In this context, specific functions could be attributed to ceramide in all steps leading to plaque formation (Figure 1). In particular, Schissel et al. demonstrated that LDL aggregation is mediated by SMase activity on sphingomyelin transported by LPs, leading to a significant increase in ceramide levels. Furthermore, they measured for the first time the amount of ceramide in lesional oxidized LDL (oxLDL), which was increased 10- to 50-fold compared with circulating LDL ceramide. Interestingly, ceramide accumulation exclusively affected lesional LDL, which was aggregated, whereas the ceramide content of unaggregated LDL remained low [114]. Recently, aggregation-prone LDL, whose levels are directly correlated with the risk of CAD, was also shown to contain enriched levels of both SM and ceramide. Aggregation-prone LDL triggers the release of matrix metalloproteinase-7 (MMP-7) from foam cells, an enzyme that may be responsible for the rupture of human atherosclerotic plaques [115]. Direct causality between ceramide and LDL aggregation is also supported by evidence that pharmacological inhibition of ceramide biosynthesis with myriocin in mice reduced the proportion of ceramide and SM in LDL particles that were less prone to aggregation. As a result, mice treated with myriocin had significantly smaller atherosclerotic lesions compared with control animals [116,117].

The molecular basis of ceramide’s role in LDL aggregation has also been investigated. One proposed mechanism focused on the formation of hydrophobic domains on the LDL surface due to sphingomyelin hydrolysis mediated by SMase activity. The change in lipid composition of the LDL phospholipid monolayer, due to the loss of the polar phosphorylcholine head group of sphingomyelins, resulted in the “hydrophobic effect”. Specifically, the increase in ceramide molecules stimulated their mutual shielding from the polar aqueous environment, eventually leading to LDL aggregation [118]. Another mechanism involves apolipoprotein B100 (apoB100), the primary protein of LDL. Specifically, the increase in ceramides by SMase activity led to a conformational change in apoB100 that was critical for promoting LDL aggregation. Indeed, this ceramide-enrichment-mediated change in apoB100 structure resulted in the exposure of specific regions of the protein that are particularly susceptible to LDL particle binding. Moreover, proteolytic degradation of these particular sites significantly counteracts LDL aggregation [119].

The subendothelial retention of oxLDL in the intima represents the second step of AS. To deposit in the vascular intima, oxLDL must overcome the barrier of vascular endothelial cells in a process called transcytosis. In this context, ceramides produced in endothelial cells greatly facilitated the transcytosis of oxLDL through the vascular endothelial barrier while promoting, at the same time, their retention. Treatment with desipramine or myriocin, beyond reducing ceramide content, counteracted this process and thus reduced the accumulation of oxLDL [120]. Regarding the mechanism by which ceramide contributed to the transcytosis of oxLDL, vascular endothelial cells can internalize oxLDL mainly through their receptor Lox-1, along with several other proteins, such as caveolin-1 [121]. Both proteins are located in lipid raft domains, and ceramide could maintain the stability of these membrane structures and ultimately promote lipid-raft mediated oxLDL transcytosis [120]. Interestingly, oxLDL per se could stimulate ceramide synthesis in lipid rafts, influencing its transcytosis through a positive feedback mechanism [114]. In particular, the increase in ceramide levels was mediated by the capacity of oxLDL to directly stimulate the activity of SMase associated to ceramide-enriched microdomains [122].

### 3.3. Ceramide and Monocytes Recruitment

Ceramide has also been associated with other critical steps in atherosclerotic lesion formation, namely monocyte recruitment, adhesion to vascular endothelium, and their subsequent differentiation into macrophages (Figure 1). In particular, the treatment of monocytes with palmitate, an essential precursor of long-chain ceramide *de novo* synthesis, promoted their adhesion to vascular bifurcations, which are extremely vulnerable to AS. This effect was mediated by the increase in the expression of CD11b, the most abundant integrin subunit of monocytes. Gao et al., also demonstrated that 24 h of treatment with palmitate was sufficient to increase the expression of CD36, one of the scavenger receptors, in monocytes. Thus, after differentiation into macrophages, treated monocytes showed an altered ability to uptake oxLDL, stimulating the formation of foam cells [123]. Furthermore, analysis of cell lysates by LC-MS confirmed that palmitate-induced increased expression of CD11b and CD36 was correlated with higher cellular C16 ceramide content. Conversely, neither treatment with the monounsaturated FA oleate nor palmitate in the presence of the fumonisin B1 inhibitor was associated with an increase in ceramide content and upregulation of CD11b/CD36, suggesting that the observed effects on monocytes are mediated, at least in part, through the ceramide *de novo* synthesis pathway [123]. In this sense, treatment of LDL with SMase enhanced their uptake into macrophages, resulting in increased cholesterol ester intracellular accumulation [121]. Moreover, hydrolysis of LDL-SM directly affected the affinity of LDL for arterial proteoglycans and favored the process of LDL aggregation and fusion, which is also stimulated by SM cleavage on the surface of atherogenic LP through the action of secreted SMase (S-SMase) produced by endothelial cells and macrophages [124]. These data indicate that ceramide accumulation due to SMase activity plays a crucial role in the conversion of macrophages into foam cells, considering that aggregated LDL strongly promotes foam cell formation [125].

### 3.4. Ceramide, VSMCs and Effects on Atherosclerosis Plaques Formation

A growing number of studies also investigated the role of ceramide during the late remodeling phase of atherosclerotic lesions (Figure 1). Interestingly, ceramide could mediate apoptosis of foam cells, leading to the formation of vulnerable plaques characterized by a necrotic core, which are the leading cause of cardiovascular complications because of their extreme susceptibility to rupture and erosion [126]. In this context, Deigner et al. demonstrated that oxLDL-mediated apoptosis of human macrophages is also associated with increased aSMase expression, which generates higher intracellular ceramide concentrations [127]. However, the mechanism by which oxLDL, aSMase activity, and ceramide accumulation trigger macrophage apoptosis have not been fully elucidated. Recently, Zhao et al., described for the first time how oxLDL-induced macrophage apoptosis is due to ER stress, which in turn is stimulated by the aSMase/ceramide signaling pathway. Specifically, the generation of ceramide by aSMase led to plasma membrane reorganization with the formation of ceramide-enriched platforms that mediate the clustering of receptors and signaling molecules. These changes induced the upregulation of some ER stress sensors that eventually activated CHOP, one of the major transcription factors that trigger ER stress-mediated apoptosis [128].

The stability of atherogenic plaques is also primarily determined by vascular smooth muscle cells (VSMCs). These cells can die by both necrosis and apoptosis, depending on the extent of the damage, and this can lead to detrimental plaque cap thinning that eventually results in subsequent plaque rupture [129]. Usually, chronic VSMCs apoptosis will induce secondary necrosis, which is responsible for the formation of the necrotic core of the most unstable plaques [130].

Remarkably, ceramide affects the apoptosis of VSMCs and modulates their phenotype. Thus, the number of VSMCs in the plaque was negatively correlated with ceramide content, whereas conversely, ceramide content was positively associated with caspase-3 content in the plaque [10]. Moreover, modified LDL activated aSMase in VSMCs, thereby increasing ceramide levels, which was accompanied by activation of several signal transduction pathways involved in inflammation and apoptosis, such as p38 MAPK and JNK. In contrast, ceramide itself was able to stimulate the activation of aSMase and protein kinases, indicating a dual role of this SL. On the one hand, ceramide could act as a second messenger to propagate apoptosis signals. On the other hand, it triggers a positive feedback mechanism to enhance its formation [131].

Lesion instability, primarily due to the presence of apoptotic cells within the plaque, could also be one of the main causes for triggering various inflammatory responses. In this regard, treating human aortic SMCs with ceramide stimulated a significant release of the proinflammatory cytokine interleukin-6 (IL-6), promoting a marked inflammatory state within the endothelium and a sustained migration of inflammatory cells [10].

Apoptosis of VSMCs is also involved in another important chronic complication of AS, namely vascular calcification [132]. Moreover, oxLDL is known to promote osteogenic differentiation and calcification of VSMCs, which negatively affects the stability and burden of atherosclerotic plaques [133]. In 2013, Liao et al. demonstrated for the first time that ceramide treatment accelerates oxLDL-induced osteogenic differentiation of VSMCs and that the nSMase/ceramide pathway stimulates vascular calcification by activating the p38 MAPK signaling cascade [134]. Recently, the same group further elucidated the molecular mechanism underlying vascular calcification. Specifically, oxLDL treatment upregulated Toll-like receptor 4 (TLR4) expression in VSMCs, which was associated with increased nuclear translocation of NF-kB p65. Inhibition of NF-kB attenuated oxLDL-induced osteogenic differentiation of VSMCs, which was conversely rescued by ceramide. Thus, these data suggest that calcification of VSMCs is modulated by TLR4 activation mediated by oxLDL, which in turn triggers the NF-kB/ceramide pathway, making it in all respects a potential novel therapeutic target for vascular calcification [135]. Interestingly, the treatment of VSMCs with C2-ceramide significantly increased the expression of some osteogenic markers, such as msh homeobox (MSX2), core-binding factor a1 (CBFA1) and alkaline phosphatase (ALPL). Moreover, this ceramide effects were even higher in the presence of phosphate, which is a potent inducer of the calcification of VSMCs [136].

The pathophysiological role of ceramide in the AS process has also been investigated in vivo. Because several in vitro studies have demonstrated the direct involvement of the aSMase/ceramide pathway in promoting lipoprotein aggregation, retention, and foam cell formation, Devlin et al., developed an aSMase knockout mouse (Asm^−/−^) starting from the apolipoprotein E knockout mouse (ApoE^−/−^), the most common murine model for atherosclerosis. Notably, compared with Asm^+/+^/ApoE^−/−^ mice, these double knockout mice showed a significantly lower number of foam cells in the early atherosclerotic lesions, accompanied by a marked decrease in LDL retention in the aortic root cell lesion [137]. In addition to this line, ApoE^−/−^ mice have been widely used to study the effects of ceramide reduction on the development of AS. Notably, most of these studies used treatment with the specific serine palmitoyl transferase inhibitor myriocin (SPT) to block ceramide *de novo* synthesis. Interestingly, SPT inhibition in young ApoE^−/−^ mice significantly reduced plasma TG and cholesterol, which counteracted the formation and progression of early atherogenic lesions [138]. Moreover, administration of myriocin was sufficient to decrease plasma levels of SM and ceramide and to affect AS even in ApoE^−/−^ mice fed a high-fat/high-cholesterol diet [139].

The effect of myriocin was also studied in the regression of AS, analyzing the size and composition of preexisting plaques after treatment of adult ApoE^−/−^ mice with the molecule. The results showed that inhibition of SPT lowered plasma levels of atherogenic lipids and caused a significant reduction in the size of atherosclerotic plaques in the aortic root. In addition, myriocin also altered plaque composition by counteracting intimal macrophage accumulation and promoting collagen deposition, ultimately leading to a more stable plaque phenotype [140]. More recently, the atheroprotective potential of myriocin has been attributed to its ability to prevent lipid uptake rather than modulate cholesterol metabolism. Indeed, treatment of ApoE^−/−^ mice with myriocin significantly decreased the expression of CD36, LOX-1, and SR-A receptors in monocytes, which are responsible for nearly 90% of oxLDL uptake. Moreover, myriocin impaired inflammatory monocyte differentiation and inhibited the release of pro-inflammatory cytokines such as IL-1, IL-6, and TNF-a, which negatively affect the stability of atherosclerotic plaques. Thus, this mechanistic study demonstrated that inhibition of SPT and reduction of ceramide could ameliorate atherosclerosis through a combination of reduced foam cell formation and anti-inflammatory properties [141].

### 3.5. Ceramide Plasma Levels and CAD

Given the abundant evidence for the clear involvement of ceramides in AS pathophysiology, these molecules have also been considered as potential biomarkers and risk predictors for coronary artery disease.

Along this line, lipidomic analyzes showed that several ceramide species, particularly Cer(d18:1/16:0), Cer(d18:1/18:0), and Cer(d18:1/24:1) in plasma, could be useful prognostic markers of adverse events in CAD patients with established stable disease or high-risk acute coronary syndrome (ACS). Interestingly, Cer(d18:1/24:0) behaved differently from the others, as its levels were reduced in CAD patients [142]. Therefore, these ceramide species were used to create a ceramide risk score (CERT) based on the evaluation of the ratios between Cer(d18:1/16:0), Cer(d18:1/18:0), and Cer(d18:1/24:1) compared with Cer(d18:1/24:0) [143]. Remarkably, the predictive potential of CERT appears to be more accurate than the measurement of serum levels of low-density lipoprotein cholesterol (LDL-C), which is currently the standard procedure [142,143]. In addition, the potential of the ceramide risk score was further supported in another study in which CAD patients were followed for up to nine years. Higher plasma levels of Cer(d18:1/16:0), Cer(d18:1/18:0), and Cer(d18:1/24:1), as well as increased ratios to d18:1/24:0 ceramide, correlate strongly with the risk of cardiovascular death. Moreover, during follow-up, high ceramide risk scores were significantly associated with a higher risk of all-cause mortality in CAD patients [144]. Although the causal relationships between mortality risk and plasma ceramide levels have not been fully elucidated, the ceramide risk score is gradually being introduced into clinical practice [145].

Differential ceramide levels have also been associated with certain features of atherosclerotic plaques measured by in vivo imaging techniques, such as IVUS-VH and NIRS. In particular, increased plasma levels of ceramide correlated positively with vulnerable plaques characterized by necrotic core tissue and higher lipid core burden. In addition, the Cer(d18:1/16:0) ratio proved to be a potent predictor of cardiac severe adverse events within 1 year (MACE) after coronary angiography in patients with established CAD [146]. In contrast, Mantovani et al. recently showed that higher circulating plasma levels of plasma Cer(d18:1/20:0), Cer(d18:1/22:0), and Cer(d18:1/24:0) significantly correlated with the severity of left anterior descending (LAD) coronary artery stenosis in patients undergoing both urgent and elective coronary angiography [147] (Table 1).

## 4. Sphingosine-1-Phosphate

Sphingosine-1-phosphate (S1P) is one of several bioactive phospholipids localized in cell membranes that exert profound mitogenic and morphogenic actions [148]. S1P is produced in the blood by a reaction catalyzed by SphK1/2 in most mammalian cells and tissues, including red blood cells, platelets, and endothelial cells [149,150]. Ishimaru et al. demonstrated that SphK2 played an important role in the development of atherosclerotic plaque, whereas the absence of SphK1 did not alter the phenotype [151]. Notably, mice genetically deficient in SphK2 have larger atherosclerotic lesions than control mice [151]. Endothelial cells can synthesize and secrete a large amount of S1P, which to a large extent, leads to a high level of S1P in the blood [152,153]. S1P is presented with different carbon long-chain base lengths, the major S1P species including 16-carbon monounsaturated S1P d16:1, d17:1, d18:0, and d18:1 in mammals. As the most abundant form, d18:1 accounts for about 80% of the total S1P in plasma [154]. Similar to other major phospholipid mediators, S1P can easily act both intracellularly as a second messenger and extracellularly, as a ligand for five different high-affinity G protein-coupled receptors (GPCRs), originally known as the endothelial differentiation genes (EDG1-5), but recently renamed S1P receptors (S1PR1-5) [155,156,157]. S1PR1-3 are ubiquitously expressed, while S1PR4-5 are specifically localized in the immune and nervous system [158,159]. S1PR1 is ubiquitous and is virtually expressed in every cell line [160]. In particular, it has a peripheral effect on the immune system and significantly impacts the central nervous system and heart [160,161]. S1PR1 has been described as the most selective receptor, binding only to Gαi/0 [162]. S1PR2 inhibits apoptosis, cell proliferation, actin remodeling, and is involved in cardiac development [163,164]. It can associate with Gαi/0, Gαq, G12/13, and Gαs, but couples most efficiently with G12/13 [162,165]. S1PR3 plays an essential role in regulating the vascular tone by vasodilation, but it also protects from myocardial ischemia and coagulation during inflammatory processes [166]. It has been reported to couple with Gαi/0, Gαq, and G12/13, although a higher affinity/likelihood for association with Gαq has been observed [167]. S1PR4 is specifically expressed in hematopoietic tissue and lung, where it plays an essential role in lymphocyte signaling, megakaryocyte differentiation, and platelet formation [168]. On the other hand, oligodendrocytes, the myelinating cells of brain, mainly express S1PR5 [169]. S1PR4 and S1PR5 mediate their signaling coupling to Gαs, Gαq, and G12/13 [162,170].

### 4.1. S1P and Endothelial Dysfunction

Whereas the role of ceramide during atherosclerosis has been associated with endothelial barrier disruption and increased permeability due to the deleterious effects of ROS, recent studies suggest a central role for S1P as an effective endothelial cell agonist that induces proliferation, calcium mobilization, adhesion molecule expression, and suppression of apoptosis, thus promoting endothelial barrier integrity [171,172,173,174]. As in cardiomyocytes, S1P binds to S1PR1-3 in endothelial cells (ECs), although most S1P-mediated responses occur via S1PR1 [175]. Morphological studies have shown that adding 1 µM S1P increases transmonolayer electrical resistance (TER) in human and bovine EC and protects the barrier from the destructive effects of some edemagenic agents such as thrombin [176]. Specific downregulation of S1PR1 or S1PR3 receptor expression by antisense oligonucleotides markedly attenuated the S1P-induced increase in TER [176]. The increase in endothelial integrity was mainly associated with improved endothelial cell spreading, decreased cell contractility, and stabilization of endothelial cell junctions. Regarding spreading EC, S1P dramatically increased F-actin polymerization and myosin light chain phosphorylation and recruited S1PR1 to the caveolin-enriched microdomain (CEM), which in turn activated the PI3K signaling pathway and increased PIP3 production (130). The accumulation of PIP3 was critical for the recruitment of Akt to the membrane, which then activated Tiam1 (T-lymphoma invasion and metastasis gene 1), a specific Rho family guanine nucleotide exchange factor (GEF) that catalyzes the exchange of Rac1-GDP (inactive) to Rac1-GTP (active) [176,177,178]. After this exchange, Rac-1 activity facilitated the recruitment of cortactin to the ARP2/3-containing actin nucleation complex, which initiated the polymerization of actin filaments at the cell periphery, expanded the membrane, and spread ECs [179,180]. There is growing evidence that S1P promotes the actin-dependent spreading of endothelial cells and may promote it by suppressing cellular contractility. This is partly supported by the fact that S1P activates Rac-1, suppressing the RhoA-dependent stress fibers formation and converting cells to an epithelial phenotype [181].

In addition, S1P also affects vascular tone. Indeed, this molecule suppresses cellular contractility by modulating nitric oxide (NO), which is produced in the vascular endothelium by the endothelial isoform of nitric oxide synthase (eNOS) and diffuses to vascular smooth muscle cells (VSMCs), promoting vascular relaxation [182]. Notably, S1P treatment promoted rapid, dose-dependent, and reversible vascular relaxation in rat mesenteric arteries via a NO-dependent pathway [183]. Along this line, the increase in Akt in response to S1P induced the phosphorylation of eNOS at serine 1179, which first hydroxylated L-arginine to Nω-hydroxy-L-arginine and then oxidized Nω-hydroxy-L-arginine to L-citrulline and NO [184,185]. After release, NO causes a dramatic decrease in cytosolic Ca^2+^ concentration by inhibiting voltage-dependent Ca^2+^ channels, activating Ca^2+^-dependent potassium channels, and finally inhibiting the formation of the calcium-calmodulin-myosin light chain kinase complex in VSMCs, which promotes vasorelaxation [186]. The role of S1P in regulating NO signaling was further confirmed by inhibition of the eNOS/NO pathway with L-NAME, which resulted in a dose-dependent reduction in the sustained effects of S1P on the endothelial barrier [187].

S1P also regulates the integrity of the endothelial barrier by binding EDG-1, which leads to the activation of MAP Kinase and the stabilization of endothelial cell-cell junctions [173]. Indeed, HUVEC cells treated with 500 nM S1P showed an increase in the localization of vascular endothelial cadherin (VE-cadherin), α-, β-, and γ-catenin at cell-cell junctions. In contrast, the translocation of VE-cadherin was reduced by microinjection of oligonucleotides designed to inhibit the expression of S1P1 and S1P3 receptors [173]. Similarly, overexpression of SP1 in human embryonic kidney cells induced the formation of well-developed adherent junctions in an S1P-dependent manner [188]. In contrast, silencing of the S1P1 receptor decreased the expression of VE-cadherin and platelet endothelial cell adhesion molecule-1 (PECAM-1), promoting junctional destabilization [189]. On the basis of these data, S1P played a well-orchestrated role in maintaining endothelial barrier function, so its agonists were used to mimic and enhance these effects. Specifically, HUVEC cells treated with FTY720 at a concentration of 100 nM showed an inhibition of vascular permeability induced by vascular endothelial cell growth factor through an increase in Akt and Erk phosphorylation. They enhanced VE-cadherin localization at cell-cell contact sites [190]. In addition, human pulmonary artery endothelial cells treated with FTY720 also showed an increase in EC barrier function, measured as TER, but independent of S1PR1 ligation [191]. In this case, a decrease in S1PR1 expression did not affect the TER increase induced by FTY720. This suggests an alternative EC barrier-improving mechanism stimulated by FTY720, possibly due to the different cell models used in this study [191]. The maintenance of endothelial integrity by FTY720 has also been demonstrated in vivo in fat-fed streptozotocin-treated rats, in which a decrease in evaginations and invaginations of microvascular walls associated with increased S1PR1 and S1PR3 expression was observed after FTY720 administration [192]. The ability of S1PR1 agonists to improve EC barrier function was not limited to FTY720 and the effects of other drugs have also been studied. Specifically, treatment with two S1PR1 agonists (CYM-5442, SEW-2871) or with a novel biological chaperone of S1P (ApoM-Fc) decreased VE-cadherin degradation, which promoted the stabilization of cell-cell junctions and ultimately increased EC resistance in vitro and in vivo [193]. However, different S1PR subtypes may have different and even opposite biological effects in regulating the endothelial barrier. In this context, high glucose culture conditions induced the expression of S1PR2 but not S1PR1 and S1PR3 in endothelial cells, leading to mitochondrial apoptosis via the Akt/GSK-3β pathway [194]. The overexpression of S1PR1 and silencing of S1PR2 had a similar effect on preventing high glucose-induced EC damage, as evidenced by a decrease in reactive oxygen species (ROS) and an increase in NO [195]. These results suggest that S1PR1 and S1PR2 have opposite effects on ECs.

### 4.2. S1P and Lipoproteins

Another essential property of S1P in atherosclerosis is its ability to bind lipoproteins. In healthy human subjects, the plasma concentration of S1P ranges mostly from 0.1 to 1.2 μmol/L, whereas, in atherosclerosis, the concentration of S1P is 0.687 ± 0.266 mmol/mL [196,197]. As a blood-derived lipid, S1P is transported by high-density lipoprotein cholesterol (HDL) in about 60% of plasma [198]. Specifically, between 50 and 70% of total S1P is transported by high-density lipoproteins (HDL), approximately 30% by albumin, and less than 10% by low-density lipoproteins (LDL) or very low-density lipoproteins (VLDL) [199]. In this context, plasma apolipoprotein M (apoM), which is mainly present in HDL, interacted directly with S1P [200]. To confirm the crucial role of apoM, HDL-S1P levels were measured in apoM-deficient mice. The results showed that the absence of apoM led to a dramatic decrease in S1P in HDL, confirming that only HDL containing apoM has S1P [200]. Although this is still unclear, Christoffersen et al. identified a lipophilic pocket in apoM that binds and protects S1P from degradation, thereby increasing its content in plasma [200]. To investigate the relationship between apoM and S1P, apoM was overexpressed in HepG2 cells or mouse liver, resulting in an increase in S1P levels in plasma and cells and liver. These results support the hypothesis that apoM promotes the accumulation of S1P and inhibits its degradation [201]. Finally, Arkenstejin et al., demonstrated that the lipophilic pocket of apoM also provides a binding domain for other molecules, such as retinol or oxidized phospholipids, that may compete with S1P for binding [202].

Moreover, HDL exhibits its anti-atherogenic function by reducing cholesterol levels, LDL oxidation, smooth muscle cell migration, platelet aggregation, and endothelial dysfunction [203]. These functions are also mediated by S1P. For this reason, many authors believed that S1P might enhance the anti-atherosclerotic phenotype when bound to HDL. As described previously, a critical step in the development of atherosclerosis is the increase in vascular endothelial damage promoted by the oxidation of LDL (oxLDL) [34]. Recently, Zheng et al. showed that HUVEC cells mainly express S1PR1 and S1PR3 under physiological conditions. In contrast, treatment with oxLDL significantly upregulated the mRNA and protein levels of S1PR2, resulting in increased secretion of interleukin-1β (IL-1β) and TNF-α. On the contrary, exogenous administration of apoM-S1P under oxLDL conditions reversed these effects [204].

### 4.3. S1P and Monocytes Recruitment

As described above, another critical step in the formation of atherosclerotic plaques is the recruitment within arteries of monocyte-derived cells that eventually differentiate into macrophages [205]. Notably, apoptosis of macrophages promotes the development of a necrotic core that increases plaque vulnerability [206]. In this context, S1P has been attributed a dual nature that depends on binding to one of the different S1PR isoforms, which in turn activate several intracellular responses [207]. Specifically, the binding of HDL-S1P to S1PR1 or S1PR3 prevented the deleterious effects of macrophage recruitment in the arterial wall. Feuerborn et al. showed that low-density lipoprotein receptor (LDL-R)-deficient (LDL-R^−/−^) mice with elevated endogenous S1P levels had reduced monocyte recruitment, resulting in reduced atherosclerotic lesion expansion [208]. Moreover, HDL-S1P inhibited apoptosis in RAW264.7 murine macrophages by activating STAT3 signaling and survivin expression, supporting the anti-atherosclerotic role of HDL-S1P [209]. On the other hand, the binding to S1PR2 promoted the recruitment of inflammatory macrophages [210]. In this context, S1PR2^−/−^/ApoE^−/−^ double-deficient mice show decreased release of IL-18 and IL-1β, leading to impaired interstitial macrophage recruitment and reduced formation of atherosclerotic plaques [211]. Indeed, S1P acts as a chemoattractant for lymphocytes facilitating the egress of lymphocytes from lymphoid organs into the circulation and the recruitment of lymphocytes to sites of inflammation [212]. Nonetheless, further studies are needed to investigate the involvement of S1P receptors in the pathogenesis of atherosclerosis.

### 4.4. S1P and VSMCs

A third important role in the development of AS is played by the proliferation and migration of VSMCs, which have important roles in atherosclerotic plaques formation from the early stage of arterial intimal thickening to advanced atheroma [213]. Indeed, VSMCs can be activated and switched to a dedifferentiated phenotype characterized by increased proliferation, migration and extracellular matrix synthesis under the modulation of lesion environment and genetic factors [214]. These cells express different S1PR subtypes: adult medial VSMCs preferentially express S1PR2 and S1PR3, whereas pup-intimal VSMCs express S1PR1 in addition to S1PR2 and S1PR3 [215]. Tamama et al. showed that HDL-S1P reduces VSMC migration by binding S1P to S1PR2, which negatively regulates Rac activity and Rho signaling pathways and inhibits cell migration and membrane ruffling [216]. These results were also confirmed in S1PR2-deficient mice, which formed a larger neointima lesion compared with wild-type animals [217]. On the other hand, transfection of S1PR1 into adult medial VSMCs increased proliferation by activating the Erk pathway [218]. The anti-atherosclerotic response mediated by S1PR1 and S1PR3 was associated with the immunomodulation of VSMCs. Indeed, VSMCs treated with HDL-S1P showed suppression of NADPH oxidase and ROS production, upregulation of cyclooxygenase-2 (COX-2) expression, and increased prostacyclin (PGI2) release through activation of S1PR2 or S1PR3, leading to a reduction in platelet aggregation and inflammatory response [219]. In contrast, the use of siRNA against S1PR2 or S1PR3 significantly reduced the ability of HDL and S1P to upregulate COX-2 [54]. Finally, HDL and S1P inhibited the production of monocyte chemoattractant protein-1 (MCP-1) in VSMCs, thereby reducing the formation of ROS by activating S1PR1 and S1PR3 [220]. These data demonstrate the complex role of HDL-S1P in modulating the fate of VSMCs in response to activating a specific S1PR subtype. As demonstrated, most studies focused on the mechanisms of VSMC phenotypic transition, whereas the detailed mechanism of their regulation of phagocytosis is less clarified. Along this line, it has been recently demonstrated that VSMCs function as phagocytes engulfing eryptotic red blood cells (RBCs), binding phosphatidylserine (PtdSer) present on their external membrane [221]. PtdSer was recognized on VSMCc by MGF8, a secreted protein acting as a bridging molecule, which in turn was bound by integrin αVβ3 receptor [221]. In addition, S1P/S1PR2 signaling has been shown to promote erythrophagocytosis and release of MFG -E8, which increased Vascular Endothelial Growth Factor-a (VEGF-a) and supported angiogenesis [221]. In particular, JTE-013, an inhibitor of S1PR2, is the only molecule that induces a decrease in RBC uptake compared to other molecules directed to inhibit S1PR1 and S1PR2 [221].

### 4.5. S1P Analogs and Effects on Atherosclerosis Plaques Formation

Based on the anti-atherosclerotic function of S1P demonstrated in vitro and in vivo, the effects of treatment with FTY720 on the development of atherosclerosis were examined in vivo in LDL-R^−/−^ and ApoE^−/−^ mice. Specifically, LDL-R^−/−^ mice fed a high-cholesterol Western diet and treated with intraperitoneal administration of FTY720 showed a dose-dependent reduction in atherosclerosis plaque formation and a reduction in the release of pro-inflammatory cytokines such as TNF-1α and IL-6 [222]. Along this line, ApoE^−/−^ mice fed a high-cholesterol Western diet and treated with oral administration of 1.25 mg/kg FTY720 for 16 weeks showed a strong reduction in atherosclerotic plaques, collagen content and macrophage migration [223]. However, in ApoE^−/−^ mice fed a normal diet, no effect of FTY720 on the development of atherosclerosis was observed, supporting the hypothesis that the atheroprotective effects of FTY720 are relevant only in a background of preexisting chronic inflammation [224]. In addition to the role of FTY720, KRP203, a selective S1PR1 agonist, was also tested on LDL-R^−/−^ mice. The results showed that treatment with KRP203 strongly reduced early or advanced atherosclerotic lesions and decreased pro-inflammatory chemokines, supporting the potential contribution of S1PR1 to the regression of atherosclerosis [225]. Therefore, any functional alteration of HDL may affect its interaction with S1P and reduce its anti-atherosclerotic effect. Despite the promising results obtained by treatment with FTY720, no information is available on the effects of long-term increase or decrease of S1P levels on the development of atherosclerosis in vivo. Along these lines, Keul et al. recently demonstrated that pharmacological inhibition of the S1P-degrading enzyme S1P lyase with 4-deoxypyridoxine (DOP), for a period of up to 12 weeks, accelerated the development of atherosclerosis and resulted in a predominantly unstable plaque phenotype and frequent plaque ruptures with atherothrombosis in an animal model of cholesterol-fed ApoE^−/−^ mice [226]. However, whether long-term elevation of endogenous S1P is pro- or anti-atherogenic remains unclear.

### 4.6. S1P Plasma Levels and CAD

In recent decades, several studies measured the levels of circulating S1P to define its role as a prognostic marker for the development of coronary artery disease (CAD). In particular, S1P showed several anti-atherogenic properties (vasorelaxation, protection against post-ischemic inflammation, inhibition of oxidation) when bound to HDL, whereas an increase in “free” or “non-HDL-bound” S1P seemed to promote the inflammatory response [227,228,229,230]. At the same time, uptake of S1P by HDL may ‘‘buffer’’ or ‘‘neutralize’’ the potentially deleterious effects of free S1P [231]. In this context, Sattler et al., reported that plasma levels of HDL-bound S1P were lower and those of non-HDL-bound S1P were higher in patients with stable CAD than in healthy controls [232]. They also correlated non-HDL-bound S1P levels in plasma with the severity of cardiac symptoms classified by the Canadian Cardiovascular Score (CCS) [233]. The results showed that the levels of non-HDL-bound S1P increased significantly with the enhancement of CCS score compared with controls without symptoms, supporting the hypothesis that non-HDL-bound plasma S1P may serve as a novel biomarker for CAD [232,233,234]. Interestingly, patients with multivessel disease had lower HDL-bound plasma S1P than patients with 1-vessel disease, confirming a negative association between CAS severity and plasma-bound S1P levels [235]. These results support the hypothesis that HDL-bound plasma S1P may serve as a favorable prognostic factor, and its measurement could be investigated to prevent the occurrence of the development of atherosclerosis and its impact on CVD.

## 5. Conclusions and Future Perspectives

In recent years, the burden of atherosclerosis has increased exponentially. Atherosclerosis is one of the major risk factors for cardiovascular disease, which is still the leading cause of death worldwide, with an estimated 17.9 million deaths per year [236]. Based on these premises, atherosclerosis has been extensively studied to understand its underlying molecular mechanisms to reduce the prevalence of this disease. Numerous reports have identified the critical factors for the initiation and development of AS. They have provided remarkable insights into the progressive cellular events that lead to the formation of mature atherosclerotic plaques. However, despite the great efforts, the pathogenic molecular mechanisms of atherosclerosis remain to be further elucidated. In this context, sphingolipids have been recognized as emerging molecules that have specific effects on several processes relevant to the development of atherosclerosis, such as endothelial dysfunction, LDL aggregation, and foam cell apoptosis. The large number of SLs observed and identified in atherosclerotic plaques supports the notion that these bioactive molecules are closely associated with atherogenesis. In particular, ceramide and S1P are undoubtedly the best-characterized sphingolipids that may exert chimeric and often opposing functions in regulating cellular fate, including promoting AS. Because the conversion between ceramide and S1P can occur very easily, altering the delicate balance of their rheostat could either promote or counteract the progression of atherosclerosis (Table 2). Moreover, the traditional notion associating ceramide with adverse and pro-atherogenic effects and S1P with beneficial and anti-atherogenic effects should be reconsidered because these two SLs may indeed alternatively exhibit protective or progressive behaviors in atherosclerosis, depending on their structural properties, biosynthetic pathways, and specifically activated signaling cascades [237]. In this regard, while very long-chain ceramides can induce apoptosis, long-chain ceramides have been associated with TNF-α secretion and LDL aggregation, suggesting that the properties of ceramides strictly depend on the length of their sphingoid bases [117,238]. On the other hand, S1P behaves like an atheroprotective molecule because it is involved in anti-apoptotic and anti-inflammatory mechanisms [115], which are crucial for maintaining the integrity and function of the endothelial barrier [239]. However, S1P can mediate lymphocyte activation and stimulate thrombus formation, both mechanisms that promote atherosclerosis. These antithetic effects of S1P appear to be mediated by the different S1P receptors. Indeed, their differential expression and specific localization are critical for the ultimate consequences of their activation, suggesting that the complexity of the SLs system is far greater than previously known.

Because ceramide has been defined as the building block of all complex SLs, its content also affects the content of other SLs species, potentially triggering progressive activation of various intracellular signaling cascades that could alter cell physiology. Indeed, the biosynthesis and degradation of SLs are complex and ubiquitous processes characterized by a dynamic network of different enzymes that are tightly interconnected. However, while this extreme complexity allows for the fine regulation of the entire SL metabolism via reciprocal and multiple checkpoints, preventing changes in a single step of the metabolic pathway from immediately leading to severe consequences, it may also pose a significant issue for the development of new therapeutic approaches. Moreover, as mentioned above, ceramide and S1P are ubiquitous molecules expressed throughout the organism and can affect particular signaling cascades and many cellular responses in different tissues. Therefore, any approach that systemically modulates their content could cause undesirable side effects in other tissues and organs that are not among the target tissues, such as the vascular system in the case of atherosclerosis. An illustrative example is FTY720, or fingolimod, an S1P receptor modulator approved by the Food and Drug Administration (FDA) in 2010 for treating relapsing-remitting multiple sclerosis (MS). This molecule indeed has significant beneficial effects on the central nervous system by restraining lymphocytes in the lymph nodes and preventing the autoimmune response. Still, it has also been associated with several serious side effects, such as liver damage, increased blood pressure, and vasoconstriction. These adverse events appear to be related to the internalization of S1P receptor-1 due to prolonged treatment with FTY720. Consequently, developing a novel S1PR1 agonist such as KRP203 that can mimic sphingosine-1-phosphate activity without inducing the internalization of the receptor may significantly improve the therapeutic strategy [225]. In addition, whereas modulating the activation of specific types of S1P receptors may offer a targeted approach, ceramide formation can be addressed either by inhibiting its release from catabolic routes or its increased synthesis. Thus, elucidating the specific cell-dependent mechanisms involved in AS promotion is crucial for identifying therapeutic interventions.

In summary, despite these limitations, ceramide and S1P, as well as several molecules involved in their metabolism, have demonstrated their potential as novel targets to counteract atherosclerosis initiation and progression in many preclinical and translational studies. Circulating levels of these two molecules appear to be reliable biomarkers for AS, in some cases even better than the LDL/HDL ratio. However, they have not yet been introduced into standard practice. This calls for further efforts to improve understanding of the complex SL metabolism in atherosclerosis and identify new therapeutic opportunities. In particular, recent improvements in multi-omics technologies and related computational methods would greatly facilitate the analysis of the SLs network, leading to the development of the aforementioned multitarget approaches for treating AS in the near future.

## Figures and Tables

**Table 1:** Ceramide species considered prognostic markers of coronary artery disease.

Species	Structure	Function	Ref.
Cer(d18:1/16:0)	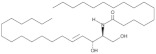	Increased circulating levels associated with higher CV mortality risk	[142,143,144]
Cer(d18:1/18:0)	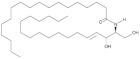	Increased circulating levels associated with higher CV mortality risk	[142,143,144]
Cer(d18:1/24:1)	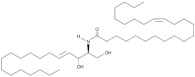	Increased circulating levels associated with higher CV mortality risk	[142,143,144]
Cer(d18:1/24:0)	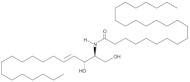	Increased circulating levels associated with lower CV mortality risk	[142,143,144]
Cer(d18:1/16:0)	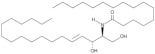	Increased plasma levels associated with higher necrotic core fraction of AS plaques and higher Major Cardiovascular Adverse Events (MACE) rate	[146]
Cer(d18:1/20:0)	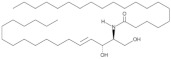	Increased plasma levels associated with higher severity of stenosis of the left anterior descending coronary artery in CAD patients	[147]
Cer(d18:1/22:0)	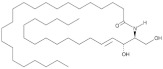	Increased plasma levels associated with higher severity of stenosis of the left anterior descending coronary artery in CAD patients	[147]
Cer(d18:1/24:0)	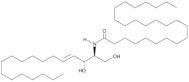	Increased plasma levels associated with higher severity of stenosis of the left anterior descending coronary artery in CAD patients	[147]

**Table 2:** Effects of the S1P and Ceramide on Atherosclerotic Plaque Formation.

Species	Target	Effects	Ref
Ceramide	Endothelial Cells	Increase of ROS production	[69,71,72,84,85,86,96,97]
		Increase in TNF-α	[87,88,89,90]
		Increase in eNOS and NO oxidation	[70,86,101,102,104,107,108,109,111]
	Lipoproteins	Increase in LDL aggregation	[114,115,116,117]
		Increase in conformational changes in ApoB100	[118,119]
		Increase in transcytosis of oxLDL	[120,121,122]
	Monocytes	Increase of CD11b and CD36	[123]
	VSMCs	Increase in apoptosis	[10,129,130,132]
		Release of proinflammatory cytokines (IL-6)	[10]
		Increase osteogenic differentiation of VSMC, leading to their vascular calcification	[132,133,134,135,136]
S1P	Endothelial Cells	Increase EC spreading by promoting F-actin polymerization and myosin light chain phosphorylation	[130,179,180,181]
		Increase vascular relaxation by modulating NO and inhibiting eNOS	[182,183,184,185,186,187]
		Increase cell contractility and VE-cadherin degradation leading to stabilization of endothelial cell-cell junctions	[173,188,189,190]
	Lipoproteins	Bound to HDL by apoM. Absence of apoM leads to a dramatic decrease in S1P	[200,201,202]
		Decrease in inflammatory state induced by oxLDL	[203,204]
	Monocytes	Inhibition of monocytes recruitment and apoptosis by activation of STAT3 via binding to S1PR1 or S1PR3	[208,209]
		Enhancement of lymphocyte recruitment to sites of inflammation via binding to S1PR2	[210,211,212]
	VSMCs	Reduction of migration by binding to S1PR2	[216,217]
		Suppression of NADPH oxidase and ROS production by binding to S1PR2 or S1PR3	[219]
		Inhibition of MCP-1 production	[220,221]
		Increase erythrophagocytosis by binding to S1PR2	[221]

## Data Availability

Data sharing is not applicable to this article.
